# Religious involvement and tobacco use in mainland China: a preliminary study

**DOI:** 10.1186/s12889-015-1478-y

**Published:** 2015-02-18

**Authors:** Zhizhong Wang, Harold G Koenig, Saad Al Shohaib

**Affiliations:** Department of Epidemiology and Statistics, School of Public Health, Ningxia Medical University, Yinchuan, 750004 China; Departments of Psychiatry and Behavioral Sciences, and Department of Medicine, Duke University Medical Center, Durham, 27705 USA; Department of Medicine, King Abdulaziz University (KAU), Jeddah, Saudi Arabia

**Keywords:** Religious attendance, Importance of religion, Tobacco use, Muslims, Mainland China

## Abstract

**Background:**

Cigarette smoking causes serious health, economic, and social problems throughout the world. Religious involvement is known to be an important predictor of health behaviors and substance use. The present study examines the correlation between religious involvements and tobacco use, and explores connections between religiosity and tobacco use in Muslims and non-Muslims in Western China.

**Methods:**

Data were examined from a representative sample of 2,770 community-dwelling adults in the province of Ningxia located in Western China. Self-report smoking, past smoking, religious attendance and the importance of religious in daily life were measured. The WHO Composite International Diagnostic Interview was used to diagnose tobacco use disorders. Three separate logistic regression models were used to examine correlations between religious involvement and smoking status.

**Results:**

In the overall sample, religious attendance was inversely associated with current smoking (p < 0.001), as was importance of religion (p < 0.05). Current smoking was also less common in those categorized as high on religious involvement. No association, however, was found between religious involvement and either past smoking or tobacco use disorders. In Muslims, both religion attendance and high religiosity were inversely associated with current smoking (p < 0.001), although no association was found in non-Muslims.

**Conclusions:**

Religious involvement is inversely related to current smoking in Western China, although this association depends on religious affiliation.

## Background

Cigarette smoking causes serious health, economic, and social problems throughout the world. Tobacco smoke is a toxic and carcinogenic mixture of more than 5,000 chemicals including nicotine, cyanide, benzene, formaldehyde, methanol, acetylene, and ammonia [1]. Smoking has major adverse effects on almost every organ system in the body, accounting for more than 10% of deaths from all causes and 30% of deaths from cancer (including 87% of lung cancer deaths) worldwide [2]. Smoking is also responsible for many other health problems include heart and blood vessel disease, stroke and cataracts [3]. According the Global Burden of Disease study, smoking causes tremendous disability, estimated at 2,276 disability-adjusted life years (DALYs) per 100,000 [4].

Cigarette smoking in China is particularly problematic because China has the largest population of smokers in the world, with over 350 million current smokers in 2012 [5]. More than 50% of males over the age of 15 smoke in China [6]. In addition, due to failed efforts to reduce or ban smoking in public places, many non-smokers (mostly children and women) also experience health problems from second hand exposure to tobacco smoke [7,8]. The number of deaths attributed to tobacco use has now reached 1.2 million per year in China, and the death toll is predicted to increase to 2 million in the near future without effective efforts to reduce smoking [9].

Smoking cessation, however, is extremely difficult because of the highly addictive nature of nicotine. Among regular smokers, withdrawal symptoms occur within two hours of the last cigarette, peaking within 24–48 hours and sometimes lasting for weeks or months [10]. Symptoms include an intense craving for another cigarette, feelings of tension and irritability, trouble concentrating, lethargy (while at the same time having difficulty falling asleep), and a decrease in motor performance, all of which drive the person to continue smoking. Thus, not only must efforts be directed at stopping people from smoking but on preventing the onset of smoking in youth. Social and cultural factors may help in this regard – particularly religion.

All major world religions place a high value on human life, and for that reason often discourage cigarette smoking, even though they may not prohibit it entirely [11]. Consequently, religious involvement is known to be an important predictor of health behaviors and substance use. Studies have found that higher religiosity is associated with a lower rate of tobacco, alcohol and illicit drug use [12-14], especially among youth [15]. In fact, a recent review of the literature concluded that greater religious involvement was associated with a lower risk of tobacco use [12]. Persons who attend religious services weekly or more have a 25% lower risk of smoking compared than those who attend less frequently. Even among those who smoke, religious attendance is inversely related to number of cigarettes smoked per day [16]. Prospective studies also find that religious involvement predicts lower rates of smoking in the future. For example, a prospective study of 4,569 individuals between ages 20 and 32 found that a higher frequency of religious attendance at baseline predicted a lower probability of both current smoking or smoking initiation during a 3-year follow-up [17]. Infrequent religious attendees are reported to be nearly twice as likely as frequent attendee to use smokeless tobacco (which is becoming more and more popular these days) [18].

Faith-based public health efforts to reduce smoking have been reported in several regions of the U.S [19]. For example, the Partnership for a Healthy Mississippi (PHM) is a program funded by churches and other faith-based organizations whose goal is to prevent youth from taking up the habit of smoking. This program has been very successful, resulting in a 25% decrease in high school students’ cigarette use. In fact, during a two year evaluation period, students’ use of any form of tobacco product dropped by 23% [20]. Similarly, another study found that the smoking cessation rate in a county where a church-based intervention was implemented was almost twice that compared to control counties within rural areas that had high concentrations of African Americans [21].

Although, researchers have shown increased interest in religion and tobacco use in recent years, there has been little research reported from non-Christian areas of the world and from developing countries [22]. Moreover, reports from studies on the effects of religious involvement on the cessation of cigarette smoking (or history of prior smoking) have not been consistent in different ethnic groups [23,24].

China has a large population with many different religious groups, including Buddhists, Taoists, Muslims, Jews, Christians, and a variety of other Chinese religions [25]. In the past 10 years, there has been a rapid increase in the percentage of Chinese who claim some type of religious practice (from 7.0% in 2001 to 23.9% in 2007) [26]. According to the Pew Research Center, over 40% of people in mainland China in the year 2010 were affiliated with at least one religion. More than seven-in-ten (73%) members of folk religions in the world and half (50%) of the world’s Buddhists live in China [27]. Also, there are over twenty million Muslims (1.8% of total population) distributed throughout China [27].

More and more research is focusing on the relationship between religious involvement and health in China [28]. For example, a large cohort study of Chinese older adults revealed that the risk of dying was 24% lower among those who frequently participated in religious activities compared to those who did not participate [29,30]. Likewise, a strong positive relationship between religious participation and subjective well-being was found in a Chinese sample, with this study showing that religion had a particularly strong effect on well-being in men [31]. Another study, this one in young Chinese women, found that higher religiosity was associated with fewer depressive symptoms and less suicidal ideation [32,33]. Little research, however, has focused on the relationship between religious involvement and cigarette smoking in China.

The present study examined the association between religious involvement and tobacco use in a large representative sample of community-dwelling adults in Western China. The objectives of this study were (1) to examine the association between religious involvement and tobacco use (current smoking, past smoking, and tobacco abuse/dependence); and (2) to explore associations between religiosity and tobacco use in Muslims compared to non-Muslims.

## Methods

### Data source and participants

Data for this study are drawn from an epidemiological study of mental disorders in the province of Ningxia located in Western China, where Muslims makes up 35% of the total population (6.4 million) [34]. Inclusion criteria were age 18 years or older and residence for at least six months or longer at the current address. Exclusion criteria were significant impairment caused by brain injury, brain tumor and/or craniotomy or dementia, being in the acute phase of a stroke, any severe illness, any obvious cognitive disabilities, or the presence of deafness, aphasia or other language barriers.

### Sample selection and procedure

Participants were identified in three stages. First, 62 primary sample units (PSU) were selected from 2,209 villages and 393 neighborhood communities using a probability proportionate to size (PPS) method [35]. Second, depending on the total number of households in the selected PSU, 60 to 210 households were systematically identified from each PSU resulting in a total of 6,890 households being selected. Third, interviewers visited sample households and used a Kish selection table [36] to identify one eligible participant from each households. A total of 6,476 participants were approached to conduct a face-to-face interview. 414 households were not found participants because no one could be located during the period of study.

Subsampling was used in most surveys to reduce respondent burden by dividing the interview into two stages. In the Stage I interview, which was administered to all respondents, information was collected on demographics and tobacco use. The World Health Organization Composite International Diagnostic Interview (WHO-CIDI) used for mental disorders assessment during this stage. A total of 5,810 participants (89.7%) completed the Stage I interview. Stage II included assessments of risk factors, services sought, religious involvement, and identification of additional disorders that were either of secondary importance or were too time consuming to assess in the full sample. A computer program was used to select participants who completed Stage I interviews to take part in the Stage II survey. The program which divided respondents into three groups based on their Part I responses. First, all respondents who (1) met lifetime criteria for at least one mental disorders assessed in Part I, or (2) met sub-threshold lifetime criteria for a mental disorders and sought treatment for it at some time in their life, were selected to complete Part II of the evaluation. Second, a probability sample was selected of 59% of respondents who did not meet criteria for membership in the first group, but gave responses in Part I indicating that they (1) ever met subthreshold criteria for Part I disorders, or (2) ever sought treatment for any emotional or substance abuse problem, or (3) ever had suicidal ideation, or (4) used psychotropic medications in the past 12 months to treat emotional problems. Third, a 25% random sample of respondents without mental disorders or emotional problems was selected to receive the Part II evaluation [37]. The present study consisted of 2,770 participants who completed the Stage II interviews.

Face-to-face computer assisted personal interviews (CAPI) [38] were carried out by lay interviewers from Ningxia Medical University. Interviewers were trained in a 7-day session by our research team. The training covered general interviewing techniques, review of the questionnaire, post-interview editing, and in- and out-of-classroom exercises. Ninety trainees passed the final test and were selected as interviewers, forty-one of them are male, and forty-nine of them are female. These interviewers then were sent into the field to administer the survey. The survey was designed as anonymous. The potential risks and benefits of the survey were described by the interviewer and the participant was asked to provide their consent by checking a box on computer screen with the response (1 = I agree to participate in the study; 5 = I do not agree to participate in the study). If the response was “I do not agree”, the CAPI program was immediately terminated automatically. The institutional review board of the Ningxia Medical University approved the study.

### Dependent variables

Cigarette smoking was assessed in terms of (1) current smoking, but without a tobacco use disorders, (2) past history of smoking (but not current use), and (3) tobacco use disorders (tobacco abuse/tobacco dependent). Smoking was measured by asking, “Are you a current smoker, ex-smoker, or have you never smoked?” here we defined the smoking as “lasting at least two months when you smoked at least once per week”. ICD-10 of Tobacco Use Disorders were diagnosed using the WHO-CID, a structured diagnostic interview that is widely used to identify mental disorders in community populations [39]. A Chinese version of the CIDI was produced by translating and back-translating the English version using the standard WHO protocol. Culture adaptation and modification research have verified the validity of this version [40].

### Independent variables

#### Religious involvements

Religious involvement was determined using measures of religious importance, attendance, and affiliation. Religious importance was measured with a single question that asked, “In general, how important are religious or spiritual beliefs in your daily life?” Responses options ranged from *not at all important* (1) to *very important* (4). Religious attendance was assessed using a single question that asked, “How often do you usually attend religious activities?” Responses ranged from *never* (1) to *more than once a week* (5). Finally, religious affiliation was determined by asking, “What is your religious preference?” Religious affiliation was coded for analysis into four categories: 1 = none, 2 = Chinese religion (i.e., Buddhist, Daoist, etc.), 3 = Western religion (i.e., Christian, Catholic, etc.), and 4 = Muslim.

Participants were divided into high and low religiosity in the following manner. High religiosity was defined as (1) attending religious activities at least 2–3 times per month and (2) indicating that religious or spiritual beliefs were very important in daily life. All other participants were placed in the low religiosity category.

### Health variables

Anxiety disorders and mood disorders were assessed using the WHO-CIDI. Anxiety disorders include agoraphobia, generalized anxiety disorder, obsessive-compulsive disorder, panic disorder, social phobia, specific phobia, and neurasthenia. Mood disorders assessed were unipolar depressive disorder and bipolar disorder.

Physical health characteristics assessed were overall self-rate physical health (good vs. poor), self-rated chronic body pain (yes vs. no), type II diabetes (yes vs. no), and hypertension (yes vs. no).

### Socio-demographic variables

Socio-demographic information collected included age, gender, education, marital status (married vs. unmarried), residence (rural vs. urban), ethnicity (Han vs. Hui), experience of migration from other areas of China (yes vs. no), and geographical region (developed vs. undeveloped).

### Statistical analyses

Analyses were performed using the Statistical Analysis System (SAS) 8.2 software (SAS Institute Inc). Differences in socio-demographic, physical, mental, religious, and smoking characteristics between males and females were examined using the Student’s t-test for continuous variables and the chi-square statistic for categorical variables. Differences by demographic characteristics, physical and mental health, and religious group and level of involvement across the three smoking categories (current, past, smoking disorder) were examined using one-way-analysis of variance (ANOVA) for continuous variables and the chi-square statistic for categorical variables. Three separate logistic regression models were used to examine correlations between religious involvement and smoking status. The final logistic regression model was then repeated in males to compare Muslims and non-Muslims (smoking exposure rate in females was too low for this comparison). Unstandardized beta and standard errors were calculated for all models. Given the exploratory nature of these analyses, statistical significance was set at 0.05 without correction for multiple comparisons.

## Results and discussion

### Sample characteristics

Demographic, health, and religious characteristics of the sample are presented in Table 1, along with a comparison of characteristics between males and females. The average age of participants was 44.2 years (SD 15.1) with a range from 18 to 89. The average education level was 5.9 years (SD 4.9) with a range from 0 to 17. The majority were female (62.4%) and from developing areas (61.9%). Between one-third and one-half (41.6%) reported their overall physical health as poor or very poor; 14.3% reported having hypertension; and 3.7% reported having type II diabetes. Lifetime anxiety disorder was present in 25.4% of the sample, whereas lifetime mood disorder was present in 5.0%, and 47.0% reported insomnia within the past year.

**Table 1:** **Characteristics of the sample**

	**Total n = 2770**	**Male n = 1042**	**Female n = 1728**
**Demographics**			
Age, years, mean(sd)	44.2 (15.1)	45.2 (15.7)	43.7 (14.8)*
Education, years, mean(sd)	5.8 (4.9)	6.9 (4.7)	5.5 (5.0)***
Ethnic, Hui,% (n)	1,150 (41.5)	433 (41.5)	717 (41.5)
Marriage, married,% (n)	2,441 (88.1)	913 (87.6)	1528 (88.4)
Region, developed,% (n)	1,055 (38.1)	402 (38.6)	653 (37.8)
Migrant, yes,% (n)	857 (30.9)	295 (28.3)	565 (32.5)*
Urban/rural, rural,% (n)	2,048 (73.9)	787 (75.5)	1261 (72.9)
**Physical health**			
Overall physical health, poor,% (n)	1,152 (41.6)	472 (45.3)	680 (39.3)**
Type II diabetes, yes,% (n)	103 (3.7)	38 (3.6)	65 (3.7)
Hypertension, yes,% (n)	396 (14.3)	144 (13.8)	252 (14.5)
Physical pain, yes,% (n)	1,411 (50.9)	464 (44.5)	947 (54.8)***
**Mental disorders**			
Anxiety disorders, yes,% (n)	705 (25.4)	212 (20.3)	493 (28.5)***
Mood disorders, yes,% (n)	140 (5.0)	47 (4.5)	93 (5.3)
Insomnia, yes,% (n)	1,302 (47.0)	466 (44.7)	836 (48.3)
**Religious involvement**			
Religious attendance, mean (SD)	1.9 (1.4)	2.1 (1.5)	1.8 (1.3)***
Importance of religion, mean (SD)	2.6 (1.2)	2.6 (1.2)	2.6 (1.2)
High religiosity,% (n)	491 (17.7)	238 (22.8)	253 (14.6)***
**Religion affiliations**			
Buddhist/Taoist ,% (n)	246 (8.9)	99 (9.5)	147 (8.5)
Muslim,% (n)	1103 (39.8)	409 (39.2)	694 (40.1)
Christian/Catholic,% (n)	54 (1.9)	23 (2.2)	31 (1.8)
No affiliation,% (n)	1367 (49.3)	511 (49.0)	856 (49.5)
**Smoking variables**			
Current smoking, yes,% (n)	489 (17.6)	466 (44.7)	23 (1.3)***
Past smoker, yes,% (n)	179 (6.4)	156 (14.9)	23 (1.3)***
TUD, yes,% (n)	26 (0.9)	25 (2.4)	1 (0.1)***

*p < 0.05; **p < 0.01; ***p < 0.001 (comparison is between male and female participants); TUD: ICD-10 of Tobacco Use Disorders.

Nearly half (49.3%) of the sample indicated no religious affiliation, whereas 39.9% were Muslim, 8.9% were associated with Chinese religions, and 1.9% were Christian. Nearly one in five respondents (17.7%) met criteria for high religiosity, with 22.8% of males doing so compared to 14.6% of females. Of the total sample, 489 (17.6%) were current smokers, 179 (6.4%) were past smokers, and 26 (0.9) met the ICD-10 of tobacco use disorders.

Comparing males and females, females were younger, had less education, and participated less frequently in religious activities (p < 0.001). Females also reported more physical pain, and were more likely to have an anxiety disorder (p < 0.001). Only 1.3% of females were current smokers, 1.3% past smokers, and one female (0.1%) met the criteria for tobacco use disorder.

### Bivariate associations

Table 2 displays associations between participant characteristics and tobacco use. Participants with less education, living in developed regions, Han ethnicity, and those with poorer physical health were more likely to be current smokers. Older participants, those of Han ethnicity, and those with hypertension or type II diabetes were at higher risk of being a past smoker. Finally, lower level of religious involvement was associated with an increased risk of current smoking, although no association was found with tobacco use disorder. Participants without a religious affiliation were at higher risk of being a past smoker (p < 0.05).

**Table 2:** **Smoking status by participant characteristics (N = 2,770 for all comparisons)**

	**Current smoking** ***Chi*** ^***2***^ ***/F value***	**Past smoking** ***Chi*** ^***2***^ ***/F value***	**TUD** ***Chi*** ^***2***^ ***/F value***
**Demographics**			
Age, years (older)	0.51	67.70***	2.96
Education, years (higher)	38.85***	0.22	0.04
Marriage (married)	7.75**	1.03	3.53
Region (developed)	2.13	0.67	2.76
Ethnic (Han vs. Hui)	36.83***	5.76*	0.10
Migrant (no)	18.86***	7.50**	0.19
Urban/rural (rural)	0.25	2.70	0.99
**Physical health**			
Overall health (poor)	7.80**	0.48	2.32
Type II diabetes (yes)	0.70	6.71**	1.15
Hypertension (yes)	1.60	7.50**	5.83*
Any physical pains (no)	11.54***	1.11	1.18
**Mental health**			
Anxiety disorder (present)	19.35***	4.20*	3.93*
Mood disorder (present)	0.71	0.11	0.38
Insomnia (present)	1.39	1.48	3.55*
**Religious involvement**			
Religious affiliation (no)	19.83***	3.83*	0.21
Religious attendance (seldom)	19.50***	0.06	0.16
Importance of religion (less)	30.71***	1.16	0.01
High religiosity (no)	19.31***	0.43	0.04

( ) characteristic associated with higher risk of smoking.

*p < 0.05, **p < 0.01, ***p < 0.001; TUD: ICD-10 of Tobacco Use Disorders.

As shown in Table 3, Muslims were less likely than non-Muslims to be current smokers, and this was especially true in males. The findings were similar among females, although current smoking rates were much lower. Muslim females were both less likely than non-Muslim females to currently smoke (p < 0.05) and were less likely to be past smokers (p < 0.001). There was no significant difference between the Muslims and non-Muslims in tobacco use disorder.

**Table 3:** **Smoking status by gender and ethnic group**

**Group**	**Current smoking N (%)**	**Past smoking N (%)**	**TUD N (%)**
Non-Muslim male, n = 609^a^	327 (53.6)***	98 (16.0)	15 (2.4)
Muslim male, n = 433	139 (32.1)	58 (13.3)	10 (2.3)
Non-Muslim female, n = 1,011^b^	19 (1.8)*	22 (2.1)***	1 (0.1)
Muslim female, n = 717	4 (0.5)	1 (0.1)	0 (0.0)
Total sample, n = 2770	489 (17.6)	179 (6.4)	26 (0.9)

^a^Non-Muslim male vs. Muslim male.

^b^Non-Muslim female vs. Muslim female.

*P < 0.05, ***P < 0.001.

TUD: ICD-10 of Tobacco Use Disorders.

**Figure 1:**
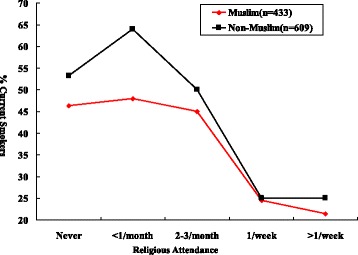
**Current smoking by religious attendance in males.**

Figure 1 present the current smoking prevalence by religious activities attendance, among Muslim males who never attended religious activities, 46.3% (19 of 41) were current cigarette smokers, compared to 21.4% of Muslim males (33 of 154) who attended religious activities more than once a week. It is also true in the Non-Muslim males, the rate of smoking for those who attended religious activities once/week or more was only 25.0% (2 of 8), accordingly, those participants they never attended religious activities with a smoking prevalence of 53.2% (290/545).

### Multivariate analyses

In Model 1, without controls, an inverse association was found between all indicators of religious involvement and current smoking (p < 0.001) (Table 4). Even after controlling for demographic variables (Model 2), and physical and mental health variables (Model 3), the relationships between religious attendance (beta coefficient is −0.43), high religiosity (beta coefficient is −1.11) and current smoking persisted (p < 0.001). Although the association between religious affiliation and current smoking disappeared when demographic variables were controlled, the relationship with importance of religion persisted in Models 2 and 3 (beta coefficient is −0.15) (p < 0.05). There were no significant associations between religious variables and past smoking or tobacco use disorder in any of the three models.

**Table 4:** **Multivariate models of religiosity and tobacco use (entire sample)**

	**Model 1** ***B (SE)***	**Model 2** ***B (SE)***	**Model 3** ***B (SE)***
**Current smoking**			
Religious attendance	−0.17 (0.03)***	−0.31 (0.06)***	−0.43 (0.25)***
Model R-square	0.01	0.30	0.30
Importance of religion	−0.21 (0.03)***	−0.15 (0.06)*	−0.15 (0.06)*
Model R-square	0.01	0.30	0.30
High religiosity	−0.67 (0.15)***	−1.10 (0.20)***	−1.11 (0.21)***
Model R-square	0.01	0.30	0.30
Religion affiliation	−0.44 (0.10)***	−0.06 (0.18)	−0.04 (0.18)
Model R-square	0.01	0.30	0.30
**Past smoking**			
Religious attendance	0.01 (0.05)	0.12 (0.08)	0.13 (0.08)
Model R-square	0.00	0.07	0.08
Importance of religion	−0.06 (0.06)	0.08 (0.08)	0.09 (0.08)
Model R-square	0.00	0.07	0.08
High religiosity	0.12 (0.19)	0.34 (0.27)	0.36 (0.27)
Model R-square	0.00	0.07	0.08
Religion affiliation	−0.30 (0.15)	−0.03 (0.23)	−0.01 (0.23)
Model R-square	0.00	0.07	0.08
**ICD-10 of Tobacco Use Disorders**			
Religious attendance	−0.05 (0.14)	−0.28 (0.20)	−0.34 (0.21)
Model R-square	0.00	0.02	0.02
Importance of religion	0.01 (0.15)	0.10 (0.21)	−0.09 (0.21)
Model R-square	0.00	0.02	0.02
High religiosity	0.10 (0.50)	−0.32 (0.64)	−0.39 (0.64)
Model R-square	0.00	0.02	0.02
Religion affiliation	−0.18 (0.39)	−0.22 (0.61)	−0.30 (0.62)
Model R-square	0.00	0.02	0.02

Model l = religious variable; Model 2 = Model l + demographics; Model 3 = Model 2 + physical health + mental health.

B = beta, SE = standard error.

*p < 0.05, ***p < 0.001.

N = 2,770 for all models.

When multivariate models were repeated in males (rates of smoking were too low among females for such an analysis), all indicators of religious involvement except religious affiliation were inversely associated with current smoking after controlling for demographics, physical and mental health, although again no associations were found with past smoking or tobacco use disorder (Table 5). When analyses were stratified by religious affiliation, inverse relationships with current cigarette smoking were found only in Muslim males.

**Table 5:** **Multivariate models examining relationships between religiosity and tobacco use in males**

	**Total male** ^**#**^ **n = 1,042** ***B (SE)***	**Muslim n = 433** ***B (SE)***	**Non-Muslim n = 609** ***B (SE)***
**Current smoking**			
Religious attendance	−0.34 (0.07)***	−0.31 (0.07)***	−0.08 (0.16)
Model R-square	0.10	0.08	0.03
Importance of religion	−0.19 (0.07)**	−0.28 (0.15)	−0.13 (0.07)
Model R-square	0.08	0.06	0.03
High religiosity	−1.22 (0.21)***	−0.97 (0.23)***	−1.56 (1.13)
Model R-square	0.11	0.09	0.03
Religion affiliation	−0.01 (0.19)	−0.12 (0.47)	−0.02 (0.20)
Model R-square	0.08	0.05	0.03
**Past smoking**			
Religious attendance	0.09 (0.09)	0.06 (0.10)	0.25 (0.19)
Model R-square	0.02	0.02	0.02
Importance of religion	0.08 (0.09)	0.02 (0.22)	0.09 (0.10)
Model R-square	0.02	0.02	0.02
High religiosity	0.27 (0.28)	0.25 (0.31)	0.49(1.14)
Model R-square	0.02	0.02	0.02
Religion affiliation	−0.16 (0.261)	−0.17 (0.61)	−0.12 (0.29)
Model R-square	0.02	0.02	0.02
**ICD-10 of Tobacco Use Disorders**			
Religious attendance	−0.37 (0.21)	−0.38 (0.23)	−0.63 (0.82)
Model R-square	0.02	0.04	0.04
Importance of religion	0.13 (0.22)	−0.08 (0.52)	0.10 (0.24)
Model R-square	0.02	0.03	0.04
High religiosity	−0.47 (0.64)	−0.64 (0.68)	−12.87 (793.6)
Model R-square	0.02	0.03	0.04
Religion affiliation	−0.24 (0.64)	11.65 (433.4)	−0.60 (0.82)
Model R-square	0.02	0.03	0.04

Model = religious variable + demographics + physical health + mental health.

B = beta, SE = standard error.

**p < 0.01, ***p < 0.001.

^#^Tobacco use in females was too low to provide stable estimates.

## Discussion

### Main findings

To our knowledge, this is the first study to examine the relationship between cigarette smoking and religious involvement in China. Religious involvement, especially frequent religious attendance and high overall religiosity were inversely related to current cigarette smoking, for those who attend religious activities more frequent likely had lower risk of smoking. And these relationships were particularly strong in males overall and Muslim males in particular, the regression model showed a minus beta coefficients value. Among Muslim males who never attended religious activities, 46.3% were current cigarette smokers, compared to 24.5% of those who attended religious activities once/week or more. In fact, even among non-Muslim males who attended religious services once/week or more, the rate of current cigarette smoking dropped from 53.2% to 25.0%. Given that over one-half of the male population of China currently smokes (52.9% overall and 63.0% of those ages 45–64), this means that there are now over 700 million male smokers in China [41,42]. Considering the negative effects that cigarette smoking has on physical health, especially in terms of chronic diseases of the pulmonary and cardiovascular systems, the findings in the present study have major public health importance.

With improvements of health care in China, its massive population is rapidly growing older and older. By 2030, the population age 65 or over is projected to increase by 20% to nearly 250 million [43]. With increasing age come chronic health problems, many of which may be caused or exacerbated by cigarette smoking. As a result, the chronic disease burden in China is expected to increase 40% in by 2030 [44]. The cost of providing healthcare to Chinese people with chronic disease threatens to drain the country of financial resources that could otherwise be directed toward economic growth.

Successful control and prevention of tobacco use requires a comprehensive approach that considers a variety of biological, psychological and cultural/spiritual factors [12]. As noted earlier, there is a increasing prevalence of religious activity, and a growing acceptance by the Chinese government towards religious organizations [26,45]. Buddhism, one of the most popular traditional Chinese religions, believes that whatever damages the body or mind must be abstained from [46]. This is also true in Islam. In fact, a “fatwa” (religious ruling not specifically based on the Qur’an but felt by religious scholars to be warranted based on the context) now places a ban on cigarette smoking in Muslims [47,48]. Based on a 2014 systematic review, of 10 studies of religiosity and smoking in Muslim populations, 7 (70%) reported significantly less cigarette smoking among those who were more religious [49]. Furthermore, of eight studies that compared Muslims and non-Muslims, three (38%) reported significantly less cigarette smoking in Muslims. These findings are consistent with the findings of the present report.

With increasing openness shown by the Chinese government to religious organizations in recent years, our findings suggest that rates of smoking in China may decline. This trend of increasing religious activity in China may then have a positive impact not only on the health of its citizens, but also on the health of the economy for years to come [50]. Although we are not suggesting that religious activity be prescribed for the people of China, further research is needed to determine to what extent these sociological and cultural factors impact smoking rates in China, just as research should be done on other factors that influence behaviors related to health. We also encourage the development of faith-based interventions like enlisting religious authorities in tobacco control campaigns undertaken by WHO’s Eastern Mediterranean Regional Office [19], and the religion facilities-based reduce tobacco use and smoking initiation modeled after successful programs in the U.S [20].

### Limitations

The present study involved only a single province in Western China, one which is largely undeveloped and rural and in which the Muslim population is much higher than in other areas of China (35% vs. 2%). Therefore, the present findings should be generalized with caution to other regions of China, and further research is needed to determine whether these findings apply to other provinces, especially large urban areas in Eastern China. Additionally, as with any study based on subjective self-report, there is the potential for recall bias which could have influenced the accuracy of the data collected and affected the smoking rates in religious participants.

## Conclusions

Religious involvement was found to be inversely related cigarette smoking in a western region of mainland China with a large Muslim population. The inverse relationship between religiosity and smoking was particularly strong in Muslim males who attended religious activities more than weekly, in whom rates of smoking were only about one-half that of non-Muslim males who never attended religious services. Given the effects that cigarette smoking has on the development of chronic disease and the increasing chronic disease burden that China will face over the next several decades, the findings reported here could have major implications for public health in China.
